# The predictive value of the modified AFP model for liver transplantation outcomes in multinodular hepatocellular carcinoma patients

**DOI:** 10.1186/s12957-023-02994-y

**Published:** 2023-03-27

**Authors:** Jingrui Wang, Jiaqi Bao, Rui Wang, Jiachen Hong, Lincheng Zhang, Qingyang Que, Shengjun Xu, Yongfeng Wu, Qifan Zhan, Yuchen Liu, Jimin Liu, Shusen Zheng, Sunbin Ling, Xiao Xu

**Affiliations:** 1 Department of Hepatobiliary and Pancreatic Surgery, The Center for Integrated Oncology and Precision Medicine, Affiliated Hangzhou First People’s Hospital, Zhejiang University School of Medicine, Hangzhou, 310006 China; 2 Zhejiang University School of Medicine, Hangzhou, 310006 China; 3 Institute of Organ Transplantation, Zhejiang University, Hangzhou, 310003 China; 4 Zhejiang Chinese Medical University, Hangzhou, 310058 China; 5 Hangzhou Normal University, Hangzhou, 311121 China; 6 Department of Pathology and Molecular Medicine, Faculty of Health Sciences, McMaster University, Hamilton, ON L8S 4K1 Canada; 7 National Center for Healthcare Quality Management of Liver Transplant, Hangzhou, 310003 China; 8 Shulan (Hangzhou) Hospital, Hangzhou, 310003 China

**Keywords:** Hepatocellular carcinoma, Liver transplantation, AFP model

## Abstract

**Background:**

There is a lack of studies focusing on the benefit of liver transplantation (LT) in hepatocellular carcinoma (HCC) patients with > 3 tumors. This study aims to establish a model to effectively predict overall survival in Chinese HCC patients with multiple tumors (> 3 tumors) who undergo LT.

**Methods:**

This retrospective study included 434 HCC liver transplant recipients from the China Liver Transplant Registry. All HCC patients had more than 3 tumor nodules. Three selection criteria systems (i.e., AFP, Metroticket 2.0, and Up-to-7) were compared regarding the prediction of HCC recurrence. The modified AFP model was established by univariate and multivariate competing risk analyses.

**Results:**

The AFP score 2 and the AFP score ≥ 3 groups had 5-year recurrence rates of 19.6% and 40.5% in our cohort. The prediction of HCC recurrence based on the AFP model was associated with a c-statistic of 0.606, which was superior to the Up-to-7 and Metroticket 2.0 models. AFP level > 1000 ng/mL, largest tumor size ≥ 8 cm, vascular invasion, and MELD score ≥ 15 were associated with overall survival. The 5-year survival rate in the modified AFP score 0 group was 71.7%.

**Conclusions:**

The AFP model is superior in predicting tumor recurrence in HCC patients with > 3 tumors prior to LT. With the modified AFP model, patients likely to derive sufficient benefit from LT can be identified.

**Supplementary Information:**

The online version contains supplementary material available at 10.1186/s12957-023-02994-y.

## Background

Hepatocellular carcinoma (HCC) is a highly prevalent malignancy with a low 5-year survival rate [1]. Liver transplantation (LT) is a radical method to manage HCC patients that typically achieves better patient survival than other treatments [2]. However, LT for HCC patients is highly limited by the shortage of donors and the risk of tumor recurrence [3]. Thus, the selection of HCC patients for LT needs to be precise and stringent.

The Milan criteria (1 tumor < 5 cm or 2–3 tumors < 3 cm) have been used for more than 20 years for HCC LT candidate selection [4]. The 5-year survival after LT is more than 70% in HCC patients fulfilling the Milan criteria. In the last 2 decades, modified and expanded selection criteria have been developed, such as the UCSF criteria [5], Up-to-7 criteria [6], and others [7–11], that are associated with an acceptable risk of recurrence. However, several selection criteria, i.e., the Milan and UCSF criteria, limit the number of tumor nodules to a maximum of 3 [4, 5, 7, 12], while others, i.e., the alpha-fetoprotein (AFP), Metroticket 2.0, and Up-to-7 models, include HCC patients with more than 3 tumor nodules [6, 9, 10]. The cause of death after liver transplantation in HCC patients is not only tumor recurrence. It is valuable to construct a model that can predict the overall survival of HCC patients after liver transplantation.

For patients with more than 3 tumor nodules, LT is sometimes the only radical treatment available, which warrants testing the predictive value of existing selection criteria for HCC patients with more than 3 tumor nodules and constructing a model that can better screen patients.

## Materials and methods

### Patients and data

The patient cohorts were derived from China Liver Transplant Registry (CLTR) database data spanning January 2015 to December 2018. A total of 434 patients with multiple HCC (tumor nodules > 3) were enrolled in the study. The variables collected were age, sex, number of tumor nodules, size of the major tumor nodule, vascular invasion, preoperative AFP level, treatment before LT [i.e., transarterial chemoembolization (TACE) and radiofrequency ablation (RFA)], preoperative model of end-stage liver disease (MELD) score and HBV infection status. Overall survival was defined as the time interval from liver transplantation to either mortality or last follow-up.

Patients with HCC distant metastasis, other organ invasions, coexisting other tumor types, perioperative mortality, or incomplete essential data for analysis (tumor size, number, α-fetoprotein (AFP) level) were excluded.

Three selection criteria—the AFP, Metroticket 2.0, and Up-to-7 models—were compared regarding the prediction of tumor recurrence.

### Statistical analysis

Competing risk analysis was performed using R (cmprsk v.2.2–10) [13] according to the methodologies provided by Scrucca and Fine & Gray [14, 15]. The net reclassification improvement (NRI) rates for recurrence (events) were calculated. The number of bootstrap replicates is 1000. The hazard ratio (HR) and c-statistics are presented with 95% confidence intervals (95% CIs). Other statistical analyses were performed using the Statistical Package for Social Sciences (SPSS 23.0; IBM Corporation, Armonk, NY). All P values were two-tailed, and significance was defined as *P* < 0.05. Follow-up after transplantation was defined as the time from transplantation to tumor recurrence, death, or the last follow-up. The HCC-related survival and incidence of recurrence were computed using the Kaplan‒Meier method, and the log-rank test was used to assess differences between the curves. Cox proportional hazards models and Kaplan‒Meier graphs were used to assess the association of the abovementioned variables with overall survival. All the factors with *P* < 0.05 in competing risk analysis were further analyzed in multivariate competing risk analysis.

## Results

### Characteristics of the study population

The baseline characteristics of the study population are presented in Table 1. Among the 434 patients, 117/434 (27.0%) fulfilled score 2 of the AFP model. The 50% cutoff MELD score was approximately 15 (≥ 15, 51.8%), and the 50% cutoff tumor number was approximately 10 (> 10, 50%). HBV-infected patients accounted for 85.5% of the patients.

**Table 1:**
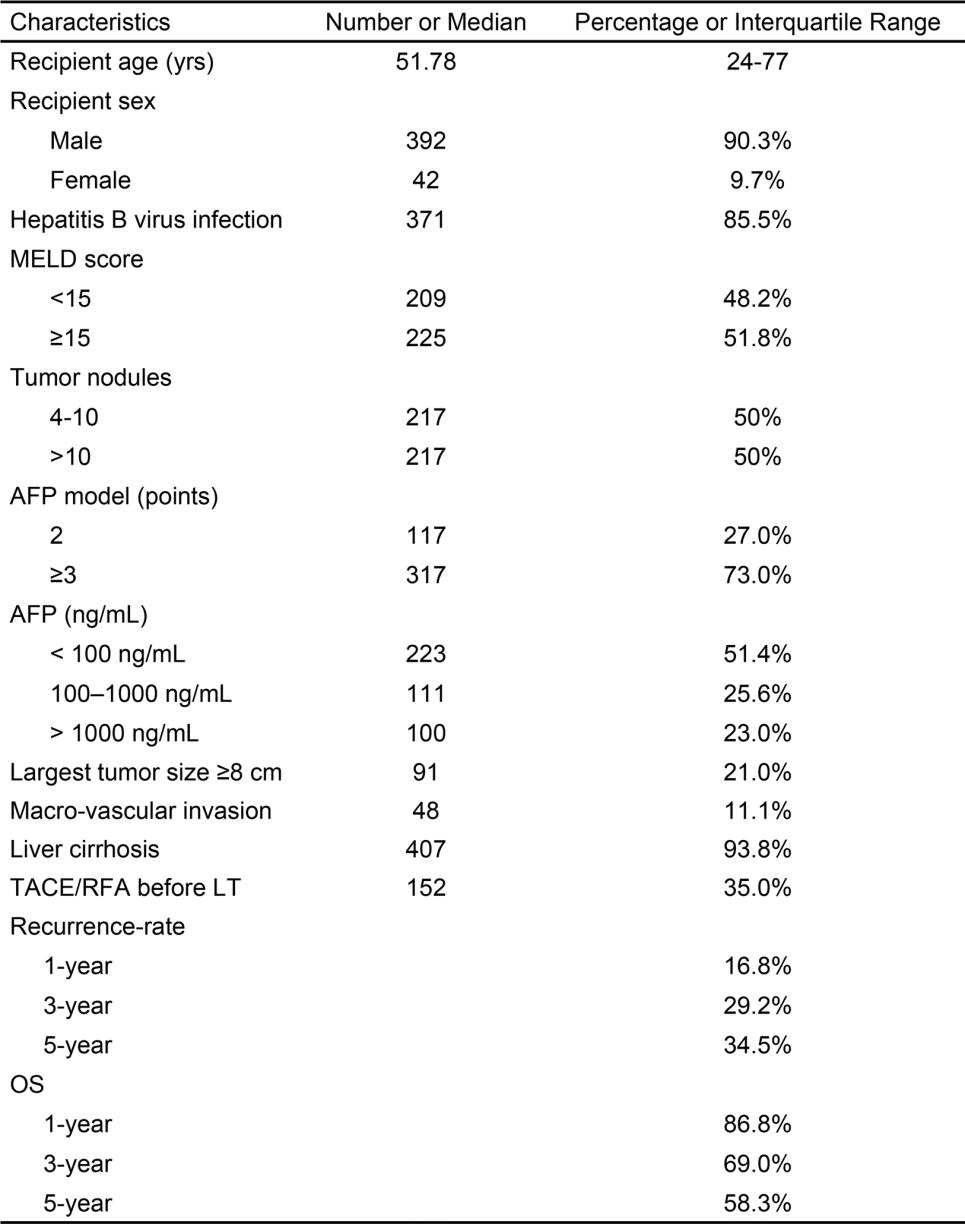
Characteristics of 434 patients after liver transplantation for hepatocellular carcinoma (tumor nodules > 3) included in the study

The median follow-up duration of the patients was 34.18 months. During this period, 119 patients developed posttransplant HCC recurrence, and 143 deaths were registered, among which 82 were HCC related and 61 were apparently non-HCC related. The rates of recurrence and HCC-related deaths were relatively high, which may be related to the high tumor burden.

### Probabilities of recurrence according to the three models

**Fig. 1:**
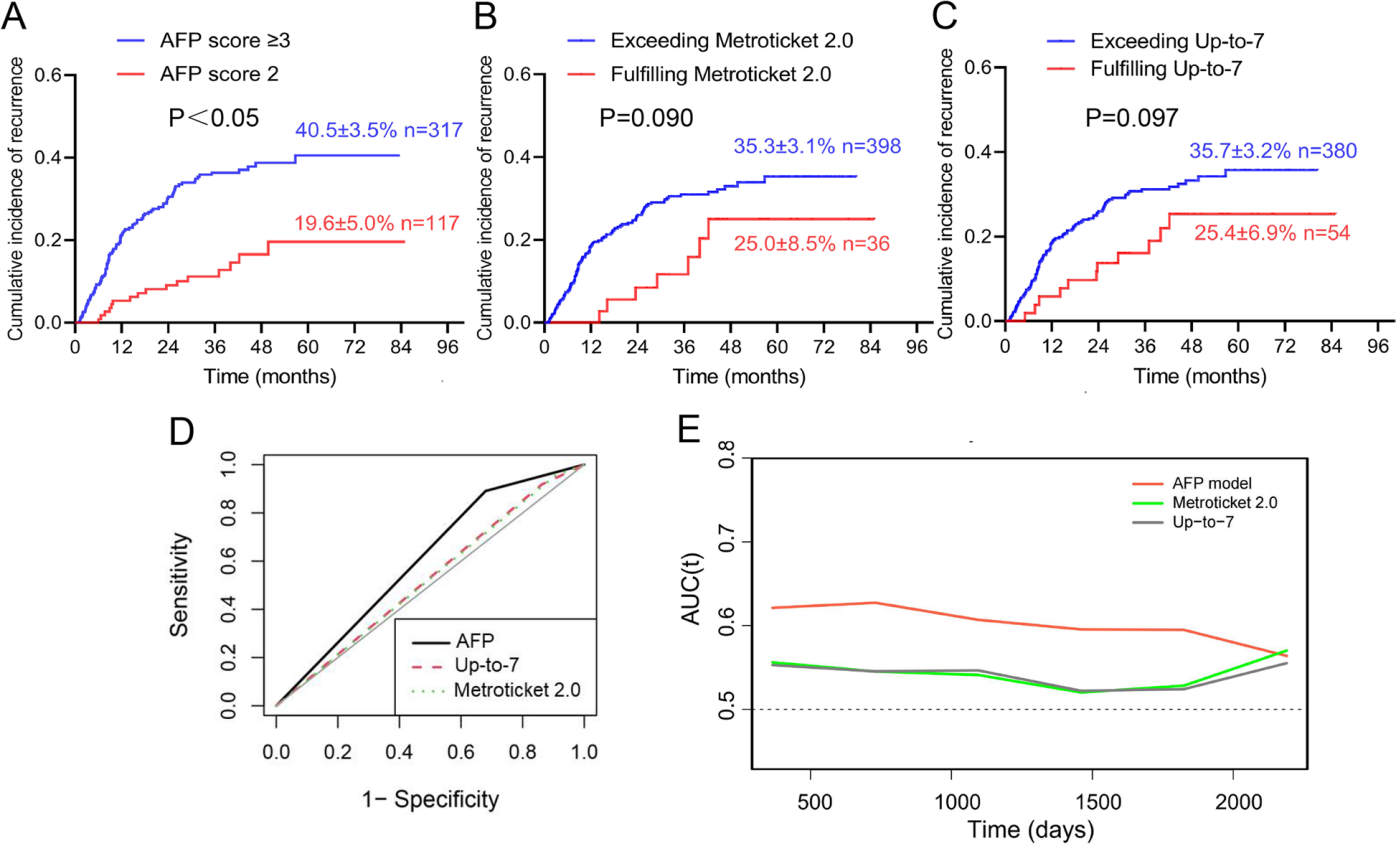
Validation and comparison of the AFP, Metroticket 2.0, and Up-to-7 models in the present HCC LT cohort. The cumulative incidence of recurrence in the present HCC LT cohort grouped by the AFP (**A**), Metroticket 2.0 (**B**), and Up-to-7 (**C**) models. Comparison of the 3 models was performed using AUC analysis and is expressed with c-statistics and 95% CI (**D**). *P < 0.05. The time-dependent receiver operating characteristic curve (ROC) value for different models according to tumor recurrence (**E**)

Regarding the AFP model, the 5-year recurrence rate in the AFP score 2 group was 19.6%, while the 5-year recurrence rate in the AFP score ≥ 3 group was 40.5% (Fig. 1A). Regarding the Metroticket 2.0 and Up-to-7 models (Fig. 1B and C), the 5-year recurrence rate in the fulfilling groups was approximately 25.0–25.4%, and the 5-year recurrence rate in the exceeding groups was 35.3–35.7%. Significantly, 27.0% (117/434) of patients were included in the AFP score 2 group, while only 8.3% (36/434) and 12.4% (54/434) were included in the fulfilling groups of the Metroticket 2.0 and Up-to-7 models, respectively (Fig. 1B and C).

### Comparison of the AFP, Metroticket 2.0, and Up-to-7 models according to net reclassification improvement

The prediction of HCC recurrence based on the AFP model was associated with a c-statistic of 0.606 (95% CI: 0.701–0.847), which was superior relative to the Up-to-7 (0.528; 95% CI: 0.496–0.559; *P* < 0.05) and Metroticket 2.0 models (0.525; 95% CI: 0.499–0.550; *P* < 0.05) (Fig. 1D). Significant NRIs were found for the Up-to-7 (0.156; 95% CI: 0.081–0.230; *P* < 0.01) and Metroticket 2.0 models (0.162; 95% CI: 0.097–0.227; *P* < 0.01) compared with the AFP score 2 model. During the entire course of the 5-year follow-up, the AFP model maintained higher ROC values than the Metroticket2.0 and Up-to-7 models (Fig. 1E). In summary, the AFP score 2 model classified the risk of tumor recurrence more appropriately than the other two models in the present cohort.

### Characteristics of patients in the AFP score 2 group

A total of 117/434 (27.0%) patients were included in the AFP score 2 group. In the present cohort, because the tumor number was greater than 3 (score 2), only the patients with AFP levels at or less than 100 ng/mL (score 0) and the largest tumor at or less than 3 cm (score 0) were included (Table S1). Interestingly, nearly half of the patients (57/117) had more than 10 tumors, indicating that if a patient expresses a low AFP level (≤ 100 ng/mL) and has tumors as large as 3 cm, a high tumor number (≥ 10) may not affect HCC recurrence.

### Analyses of risk factors for overall survival of the patients

Nine factors were associated with the analysis of the factors predicting overall survival. Using univariate and multivariate competing risk analyses, AFP level > 1000 ng/mL (hazard ratio [HR] 2.034, 95% confidence interval [CI] 1.430–2.892, *P* < 0.001), largest tumor size ≥ 8 cm (hazard ratio [HR] 2.183, 95% confidence interval [CI] 1.524–3.128, *P* < 0.001), vascular invasion (hazard ratio [HR] 1.922, 95% confidence interval [CI] 1.250–2.954, *P* = 0.003), and MELD score ≥ 15 (hazard ratio [HR] 1.443, 95% confidence interval [CI] 1.027–2.027, *P* = 0.035) were associated with overall survival (Table 2).

**Table 2:**
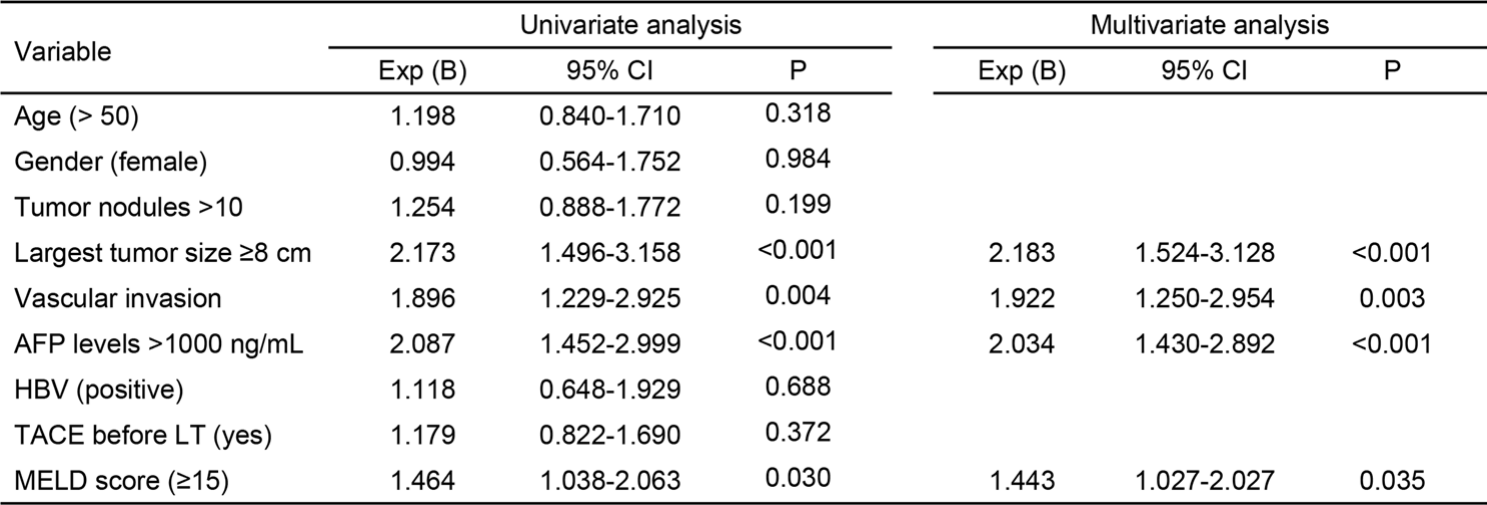
Univariate and multivariate Cox regression analysis of risk factors for overall survival of the patients

### Combining AFP level, largest tumor size, vascular invasion, and MELD score to predict the overall survival of patients

**Fig. 2:**
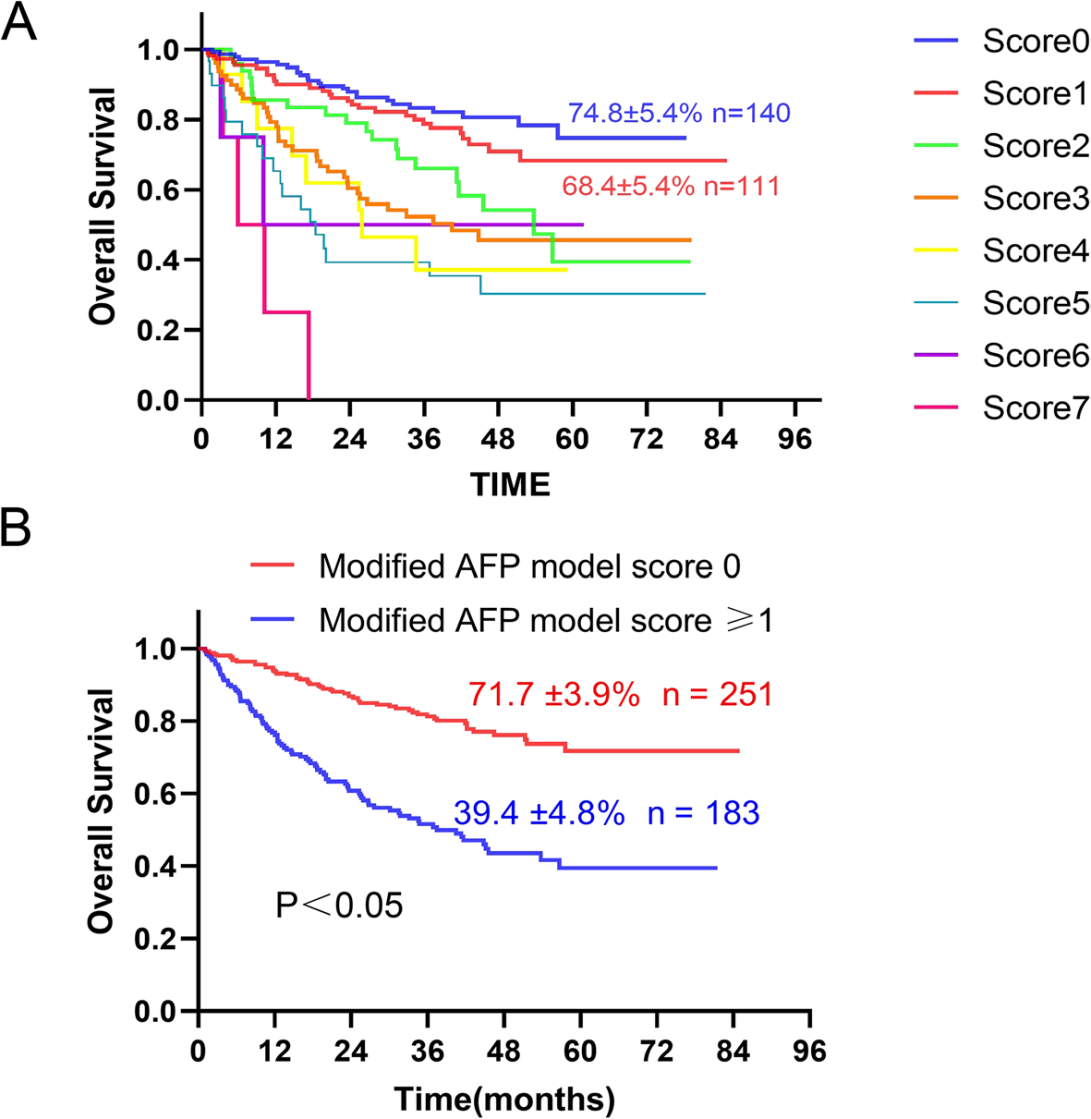
Overall survival predicted by the Four Factors score (**A**). Overall survival predicted by the Modified AFP model score (**B**)

We further combined the four factors to predict the overall survival of the patients (Table 3). Patients in the score 0 group achieved a 5-year survival rate of 74.8%, while those in the score 1 group achieved a 5-year survival rate of 68.4% (Fig. 2A). There was no significant difference between the two groups (*P* = 0.239). We considered that the MELD score was not necessary to predict OS in the low-risk group of patients. Therefore, we removed the MELD score from the four factors and built the modified AFP model (Table 4).

**Table 3:**
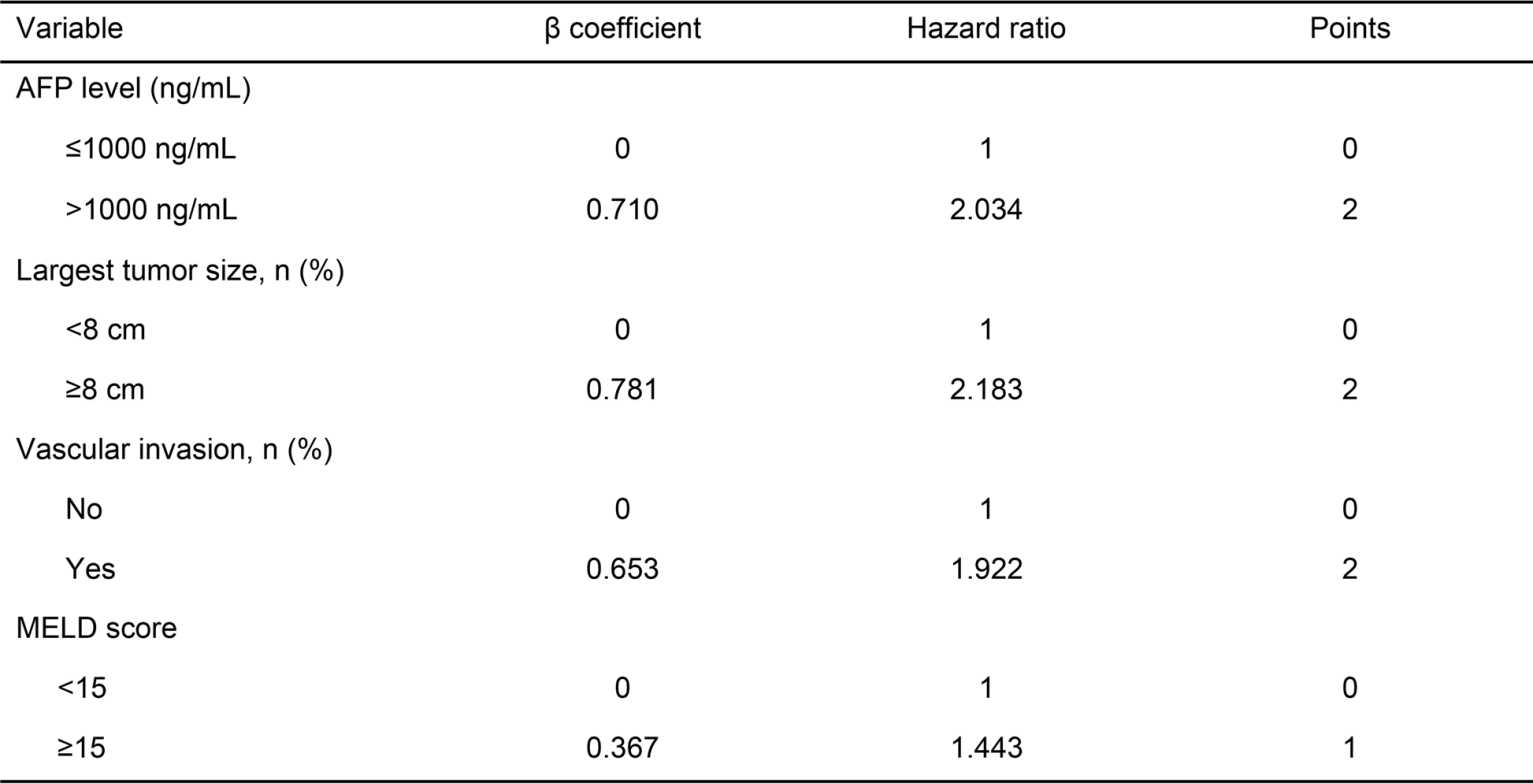
Four Factors model

**Table 4:**
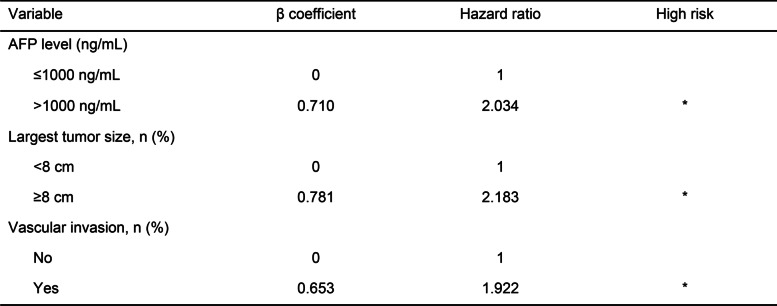
Modified AFP model

### The modified AFP model predicts the overall survival of the patients

A total of 251/434 (57.8%) patients were included in the modified AFP score 0 group (Fig. 2B). The 5-year survival rate in the modified AFP score 0 group was 71.7%, while the 5-year survival rate in the modified AFP score ≥ 1 group was 39.4% (Fig. 2B). NRI were found for the modified AFP model (0.0746; 95% CI: − 0.023–0.172; *P* = 0.13) compared with the AFP score 2 model.

Patients with AFP levels < 1000 ng/mL, largest tumor size < 8 cm, and no vascular invasion had higher overall survival rates.

## Discussion

The Milan criteria were proposed more than 20 years ago and allow a subset of HCC patients to be successfully treated by LT. In patients fulfilling the Milan criteria, the upper limit of the tumor number is only 3, indicating that patients with 4 or more tumors are not recommended as LT candidates. In the last two decades, numerous studies have attempted to expand the Milan criteria, and successful outcomes have been achieved [16]. Although several systems of criteria include patients with more than 3 tumor nodules, no study has compared or validated the value of these selection systems in this subpopulation of HCC patients, i.e., those who have more than 3 tumor nodules. For HCC patients with more than 3 tumor nodules, traditional treatments such as tumor resection or TACE can hardly achieve radical resection. Therefore, LT is sometimes the only choice for these patients. Few studies have focused on the benefit of LT in HCC patients with more than 3 tumor nodes or have discussed the selection criteria in this subpopulation. Thus, this study aimed to test the existing models in a cohort of HCC candidates from the CLTR and to establish a model that can effectively predict overall survival in all patients with more than 3 tumor nodules.

We tested three models: the AFP, Metroticket 2.0, and Up-to-7 models. Regarding the Metroticket 2.0 and Up-to-7 models, a few patients (8.3–12.4%) were included in the low recurrence risk group, and the 5-year recurrence rate was approximately 25% in both models. By comparison, 27.0% of patients fulfilled the requirements of the low recurrence risk group of the AFP model, with an acceptable 5-year recurrence rate of 19.6%. Additionally, in the cohort, the 5-year recurrence rate in the high-risk group was 40.5%. Compared with the Metroticket 2.0 and Up-to-7 models, the AFP model was less restrictive regarding the number of tumor nodules. In particular, for patients with more than 7 nodules, only the AFP model could identify low-risk recurrence patients, which explains why the AFP model included more patients in our cohort. This finding suggested that the AFP model could be used in LT candidate selection among HCC patients with more than 3 tumor nodules.

The AFP model has been verified in Western and Latin American populations [9, 17, 18]. However, in those cohorts, the median tumor number was only 2; however, in our cohort, nearly half of the patients in the total population and the AFP score 2 group had more than 10 tumors. Thus, the tumor number is not the most critical risk factor. Another difference is the HCC etiology. Most of the patients in this cohort had HBV infection, and this study confirmed the effectiveness of the AFP model in HBV-related HCC patients. Specific to the AFP score 2 group, all the patients had an AFP level < 100 ng/mL and the largest tumor size ≤ 3 cm, facilitating easy clinical application. In other words, a low AFP level and small tumor diameter may reflect less tumor biological aggressiveness, which results in better clinical outcomes after LT. HCCs with AFP scores > 2 had significantly more aggressive pathological features than HCCs with scores < 2 [17].

The AFP model focuses on the patient's postoperative tumor recurrence. We further established a model for predicting OS with Cox proportional hazards analysis. According to this model, the effect of the MELD score on patient survival was not statistically significant when the other three factors were negative. Therefore, we removed the MELD score from the four factors and built the modified AFP model. A total of 57.8% of patients in this cohort fulfilled the model requirements, with a five-year survival rate after liver transplantation exceeding 70%. The five-year survival rate after LT in this group of patients is close to that of HCC patients fulfilling the Milan criteria [2, 19]. According to the Milan criteria, this group of patients would be excluded from receiving liver transplantation. With this model, however, we can select patients from this group who would benefit from liver transplantation. Compared to the AFP model, the modified AFP model could screen more patients who could benefit from liver transplantation. However, we didn't find a significant difference by NRI index (*P* = 0.13). We considered that it might be caused by the insufficient number of cases. Significantly, more data are needed to verify this conclusion, especially in Western or other Asian countries and regions.

In conclusion, for HCC patients with multiple tumor nodules, LT is sometimes the only radical treatment, and proper selection for LT within this population is crucial for the benefit of patients and the rational allocation of donors. For the existing standard, the AFP model can effectively identify low-risk recurrence HCC patients with multiple tumor nodules. The modified AFP model allows for selecting HCC patients with multiple tumors who can derive adequate benefit from liver transplantation. However, the standard requires continuous optimization and expansion.

## Supplementary Information

**Additional file 1: Table S1.** Simplified, User-friendly Version of the AFP Model [1].

## Data Availability

The data that support the findings of this study are available from CLTR (http://www.cltr.org/) but restrictions apply to the availability of these data, which were used under license for the current study, and so are not publicly available. Data are however available from the authors upon reasonable request and with permission of CLTR (http://www.cltr.org/).

## Abbreviations

LT: Liver transplantation
HCC: Hepatocellular carcinoma
AFP: Alpha-fetoprotein
TACE: Transarterial chemoembolization
RFA: Radiofrequency ablation
CLTR: China Liver Transplant Registry
NRI: Net reclassification improvement
HR: Hazard ratio
